# The occurrence of *Chlamydia *spp. in pigs with and without clinical disease

**DOI:** 10.1186/1746-6148-8-9

**Published:** 2012-01-26

**Authors:** Stina Englund, Carl Hård af Segerstad, Frida Arnlund, Eva Westergren, Magdalena Jacobson

**Affiliations:** 1 National Veterinary Institute, 751 89 Uppsala, Sweden; 2 Stavby-Väsby 86, 747 94 Alunda, Sweden; 3 Department of Clinical Sciences, Faculty of Veterinary Medicine and Animal Science, Swedish University of Agricultural Sciences, P.O. Box 7054, 750 07 Uppsala, Sweden

## Abstract

**Background:**

Within the genera *Chlamydia*, the development of refined diagnostic techniques has allowed the identification of four species that are capable of infecting pigs. The epidemiology, clinical, and zoonotic impacts of these species are however largely unknown. The study aimed to investigate the presence of *Chlamydia *spp. in the intestines of growing pigs and in conjunctival swabs from finisher pigs, and relate the findings to clinical signs.

**Results:**

By histology, 20 of 48 pigs had intestinal lesions that may be consistent with chlamydial infection. By PCR, forty-six of the pigs were positive whereas two samples were inhibited. Sequencing of 19 DNA extracts identified these as *Chlamydia suis*. By immunohistochemistry, 32 of 44 samples were positive and a significant relationship was detected between macroscopically visible intestinal lesions and a high degree of infection. By real-time PCR, a significant difference was detected between pigs with and without conjunctivitis when a Ct value of 36 was employed but not when a Ct value of 38 was employed.

**Conclusions:**

*Chlamydia suis *was demonstrated in most samples and overall, no correlation to clinical signs was detected. However, a correlation was noted between samples with a high degree of infection and the presence of clinical signs. It is possible, that the intensive pig production systems studied might predispose for the transmission and maintenance of the infection thus increasing the infectious load and the risk for disease in the pig.

## Background

The *Chlamydiaceae *family including the genera *Chlamydia *is a well-recognised cause of disease in many animal species including cats, ruminants, birds and humans [1,2]. Within the genera, nine distinct species have been identified. Four of these have been described in pigs (*Chlamydia (C.) abortus, C. psittaci, C. pecorum*, and *C. suis*) and related to reproductive disorders, conjunctivitis, enteritis, pneumonia, polyarthritis, pleuritis and polyserositis [1,3-5]. The epidemiology, clinical, and zoonotic importance of these species are however largely unknown [6-8]. However, *C. suis *seems to be both common and widespread, often occurring in mixed infections with *C. abortus *and *C. pecorum *[2].

In gnotobiotic pig challenge studies from USA, *C. suis *caused dose-dependent diarrhoea in young piglets. Histologically, villi atrophy, tip erosions, necrosis, inflammatory changes and lymphangitis were noted in the distal small intestine. Chlamydial antigens were demonstrated in enterocytes and chlamydiae were re-isolated from tissue specimens and faecal swabs [9,10]. However, weaned pigs developed microscopical lesions but remained clinically healthy [11]. Similar lesions have also been demonstrated in clinical cases of diarrhoea in weaned pigs [12]. However, other studies have not been able to confirm a causal relationship [3,8].

A few studies have addressed conjunctivitis [13]. Chlamydiae were seen in eight pigs by ultrastructural examination and were isolated in two pigs [4], and subclinical conjunctivitis was experimentally induced in 3-day-old gnotobiotic piglets [14].

Isolation is the gold standard for diagnosis but many strains are difficult to cultivate [2,5]. Other methods includes serology, immunohistochemistry, histology, and PCR on faeces, mucosal swabs or tissue specimens [2]. Because of cross-reactivity, some tests may lack sensitivity and specificity [5,12,15]. In a study comparing four diagnostic methods, *C. suis *were demonstrated in 42% of the samples by PCR and in 33% by culture. The sensitivity was 94.4% and specificity 81.0%, whereas the two ELISAs performed considerably weaker. PCR was concluded to be a reliable, highly sensitive and specific tool for detection of *C. suis *[16].

The present study aimed to investigate the presence of *Chlamydia *spp in the intestines of growing pigs and in conjunctival swabs from finisher pigs, and relate those findings to the occurrence of clinical signs.

## Methods

The study was approved by the Ethical Committee for Animal Experiments, Uppsala, Sweden. All herd owners had given an informed consent prior to the study.

### Herds and animals

DNA from enteric specimens originated from a previous study on growing pigs with diarrhoea [17]. The samples originated from the major pig producing areas of Sweden, *i.e*. in the south-western and eastern parts of the country, and included 36 pigs from six herds with poor performance and diarrhoea in growing pigs, and 12 pigs from four herds with good performance and no diarrhoea. From each of the poor performance herds, three case and three control pigs were chosen, and from each of the four good performance herds, three control pigs were chosen. The case pigs had diarrhoea that had commenced within 2 days and no other diseases were evident. The control pigs were matched to the case pigs for age and sex, but showed no signs of clinical disease. The control pigs had a mean age of 72 days and a mean weight of 18.7 kg, and the case pigs had a mean weight of 12.1 kg. The pigs submitted from the good performance herds had a mean age of 67 days and a mean weight of 23.1 kg. All animals were weaned at approximately 5 weeks of age and none of the pigs had been treated with antibiotics. The pigs were transported to the laboratory and euthanized within 15 min prior to necropsy.

Further, animals from three finisher herds were sampled based on the present occurrence of conjunctivitis. The pigs originated from several piglet-producing herds and were introduced to the finisher herds at 25-30 kg. b.w., *i.e*. 4-14 weeks prior to sampling. They were kept 10 pigs per pen in units of 300 pigs each. In two herds, 7 case and 7 control pigs were selected from each of 3 units, and in one herd, 20 case and 20 control pigs from one unit were selected. The case pigs were selected based on clinical signs of moderate to severe conjunctivitis, defined as hyperaemia and chemosis with epiphora and/or muco-purulent secretion. The control pigs were selected from the same pens as the case pigs but showed no or only mild conjunctivitis, *i.e*. none to slight hyperaemia in the conjunctiva and no chemosis or epiphora.

### Necropsy and sampling

The necropsies were carried out at the Department of Pathology at the National Veterinary Institute, Uppsala, or at AnalyCen, Skara. The animals were stunned with electricity, weighed and exsanguinated, and necropsy was immediately performed. All gross lesions were noted, and specimens for histological examination were taken from ileum and from all macroscopically visible lesions. The samples were fixed in 10% buffered formalin, embedded in paraffin blocks, sectioned and stained with haematoxylin and eosin according to standard protocols.

The finisher pigs were snared and sterile cotton swabs were rubbed against the conjunctiva in the conjunctival sac in the left and right eye, respectively. The swabs were placed in sterile tubes and transported to the laboratory.

### Polymerase chain reaction

Intestinal samples were prepared from a 0.5 cm^3 ^piece of frozen mucosa from the distal ileum. DNA was extracted by phenol/chloroform and precipitated by ethanol [18,19]. DNA from the conjunctival swabs was extracted according to K. Sachse [20] and all samples were analysed by real-time PCR according to Everett and others [21]. The primers TQF and TQR targeted the 23 S ribosomal DNA and detected all members of the family *Chlamydiaceae*. Multiple negative controls were included and DNA from *Cp. abortus *and *C. suis *(kindly provided by K. Sachse, Friedrich-Loeffler-Institute, Jena, Germany) were used as positive controls.

To detect false negative PCR results due to inhibiting agents, an internal amplification control (mimic) was constructed and used as previously described [22]. The primers used in the mimic producing PCR were TQFActin (5'GAAAAGAACCCTTGTTAAGGGAGCCATGTACCCTGGCATTG-3') and TQRActin (5'-CTTAACTCCCTGGCTCATCATGGATCCACACGGAGTACTTGC-3'). The sequence of the ROX-labelled mimic probe used in real-time PCR was 5'-CCGACAGGATGCAGAAGGAGATCA-3'.

The conjunctival swabs were analysed both undiluted and diluted 1:100 in accordance with the standard protocol applied at the NVI, and a positive reaction in at least one of these dilutions was regarded as positive. In the statistical analyses, threshold values below Ct 36 were regarded as positive whereas values between Ct 36 and Ct 38 were regarded as doubtful. The results were calculated separately for the respective thresholds. Values above Ct 38 were regarded as negative or were excluded in the calculations. The difference in detection rate of *Chlamydia *spp. between pigs with moderate to severe conjunctivitis and the control pigs were analysed by chi-square test.

### DNA sequence analysis

Sequence analysis was performed on 19 samples from the intestinal specimens giving a strong positive reaction in the PCR. PCR was performed with primers 16SF2 and 23R according to Everett and Andersen [23] on DNA samples from seven case pigs and six control pigs from poor performance herds and six pigs from good performance herds. The predicted PCR product of 600 bp as well as one PCR product of larger and one of smaller size was purified prior to sequencing by using the GFX PCR DNA and Gel Band Purification Kit (Amersham Bioscience Europe, GmbH Germany). The purified products were sequenced with the same primers used in the PCR and by using the BigDye Terminator v3.1 Cycle Sequencing Kit (Applied Biosystems, Foster City, CA, USA) in combination with ethanol/EDTA/sodium acetate precipitation, according to the protocol supplied. Thermocycling was performed in a GeneAmp 2700 Thermocycler (Applied Biosystems). The sequencing products were subjected to electrophoretic separation and on-line detection on an ABI PRISM 3100 Genetic Analyzer (Applied Biosystems), followed by BLAST search.

### Immunohistochemistry

Paraffin-embedded intestinal specimens were available from 44 of the 48 growing pigs. 4-μm thick sections of the formalin-fixed, paraffin-embedded blocks where cut and placed onto positively charged slides (Polysine™, Menzel-Gläser, Braunschweig, Germany). Prior to immunostaining, deparaffinisation and hydration where done in xylene and graded ethanol to distilled water. After hydration, a blocking for endogenous peroxidase where done in 0.03% H_2_O_2 _in Tris-buffered saline (TBS) and nonspecific binding sites where blocked with 2% bovine serum albumin (BSA). Antigen retrieval was performed by heat induced epitope retrieval (HIER) in retrieval buffer pH = 9 (TBS-EDTA), using microwave as heat source. HIER was performed by heating the Polysine™-slides immersed in retrieval buffer for 7 min at 750 W followed by 14 min at 340 W. After completed heating procedure, the slides remained in the retrieval buffer at room temperature for 20 min. The slides where incubated with a mouse monoclonal antibody against Chlamydia, clone ACI (Progen Biotechnik Gmbh, Germany) diluted 1:100. Visualisation of the bound primary antibody was achieved by using Dako EnVision™ + System utilising an HCP-labelled anti-mouse monoclonal antibody (Glostrup, Denmark), and the presence of the relevant antigen was detected with 3,3'-diaminobenzidine (DAB, NVI, Sweden). Slides were weakly counterstained with haematoxylin. The staining included at least one positive and one negative control section. The positive control originated from previously confirmed routine cases. The results were graded as negative (-), sparse (+), moderate (++) or abundant (+++) occurrence.

## Results

### Necropsy

No gross lesions were observed in the pigs from the good performance herds. In the pigs from the poor performance herds, five control pigs and 20 pigs with diarrhoea had macroscopic lesions consistent with *Lawsonia (L.) intracellularis *infection and one had a parasitic colitis. By histology, lesions that may be consistent with chlamydial infection [9] were noted in 20 pigs: Villus atrophy was noted in six pigs with diarrhoea and in ten control pigs from the poor performance herds. Epithelial exocytosis or necroses were noted in five pigs with diarrhoea, in one control pig from the poor performance herds, and in one pig from the good performance herds (Table 1).

**Table 1:** The findings in intestinal specimens from growing pigs with diarrhoea (case), clinically healthy control pigs from the same poor performance herds (casecontrol), and from healthy pigs originating from good performance herds (control), examined by immunohistochemistry (IHC), necropsy, and PCR.

		Case	Casecontrol	Control
		(n = 18)	(n = 18)	(n = 12)
**IHC**	-	1 (7%)	9 (53%)	2 (17%)
	+	5 (33%)	3 (18%)	7 (58%)
	++	7 (47%)	4 (24%)	3 (25%)
	+++	2 (13%)	1 (6%)	0
	missing	3	1	-
**Necropsy**	Villi atrophy	6 (33%)	10 (56%)	0
	Villi necrosis	5 (28%)	1 (6%)	1 (8%)
**PCR**	positive	18 (100%)	18 (100%)	10 (83%)
	inhibited	0	0	2 (17%)

The percentage given within brackets is calculated on the actual number of analyses performed in each group, *i.e*. the "missing samples" are excluded

### Microbiological analyses

In the previous study [17], *Brachyspira pilosicoli *were demonstrated in eight case pigs and five control pigs, *Campylobacter jejuni*; in one case pig and four control pigs, *Yersinia enterocolitica *in three case and five control pigs, haemolytic *Escherichia (E.) coli *in two case and two control pigs, *Clostridium perfringens *in two case pigs, and *L. intracellularis *were previously demonstrated in 11 case and eight control pigs from the poor performance herds. In addition, haemolytic *E. coli *had been demonstrated in two pigs from the good performance herds. Rotavirus had been demonstrated in one case pig. Coronavirus was not included in the study, since Sweden has previously been shown to be free from transmissible gastroenteritis and porcine epidemic diarrhoea. *Balantidium coli *were demonstrated in one case and three control pigs from the poor performance herds, and in one pig from the good performance herds.

### Real-time PCR and DNA sequence analysis

Of the 48 enteric samples analysed, 46 were positive for *Chlamydiaceae *(Table 1). Two samples could not be evaluated due to amplification inhibition. The major band of 600 b.p. found in all samples was confirmed as *C. suis *in the 19 samples subjected to sequence analysis. The larger PCR product described in the Methods' section was 98-100% identical to *Escherichia coli *whereas the smaller PCR product did not match any sequence in the BLAST search.

In the PCR analyses on the conjunctival swabs, one pig with conjunctivitis and three control animals together with their matched counterparts were excluded from the statistical calculations, since the PCR was partially or totally inhibited. Thus, in total, 116 conjunctival swabs were included in the statistical analyses. Of these, three samples from pigs with conjunctivitis gave weakly positive reactions (Ct > 38 in undiluted samples) and were judged as negative.

Employing a cut-off value of Ct 36, 45 samples from pigs with conjunctivitis and 35 control pigs were determined as positive by PCR. Using a cut-off value of Ct 38, 48 samples from pigs with conjunctivitis and 42 control pigs were determined as positive. A statistically significant difference (*P *= 0.03) was detected between pigs with and without conjunctivitis when a Ct value of 36 was employed. The difference was mainly related to one of the herds (18 positive pigs with conjunctivitis and 12 positive control pigs). When a Ct of 38 was employed, no significant differences were noted.

### Immunohistochemistry

The results are shown in Table 1. Among the case pigs, 6 (40%) were negative or were carrying a low-grade infection. Among the control pigs from good performance herds, 9 (75%) were negative or sparsely (+) infected. The two control specimens in which the PCR analyses had been inhibited were graded as + and ++, respectively, by immunohistochemistry. A significant (*P *< 0.05) relationship was detected between the presence of clinical signs and a high degree (++ or +++) of infection. No relationship was detected between the occurrence of histological lesions and the demonstration of chlamydial antigen by immunohistochemistry.

## Discussion

The pig intestine is considered as the natural reservoir for *C. suis*, and the microbe seems generally to be well adapted to its host [1]. In the present study, *Chlamydia *spp. were demonstrated in high prevalence in all herds investigated. This is consistent with the few studies that have previously addressed the occurrence of *Chlamydia *spp. in pig herds. Overall, it was not possible to relate the demonstration of the microbe to the presence of diarrhoea. However, based on the immunohistochemistry, pigs with diarrhoea might have been more heavily infected than the healthy pigs. This is in consistency with the results from another study on 447 pigs submitted for necropsy, where it was not possible to relate the presence of *Chlamydia *spp. to the occurrence of diarrhoea, but in 12 cases, it was the only pathogen found [6]. Similar experiences have also been noted in calves and sheep [24-26].

In the study by Becker *et al. *[13], *C. suis *was significantly (*P *< 0.0001) related to conjunctivitis in extensive pig production systems, whereas in intensive farming systems, high prevalences (88-90%) were found in both pigs with conjunctivitis and in clinically asymptomatic pigs. This is in accordance with the findings in the present study. Becker *et al. *discussed, that environmental factors might predispose to infection. However, in one of the herds in the present study, factors such as emission of ammonium and carbon dioxide gases, air movements and overcrowding were investigated, but it was not possible to relate any environmental factor to the occurrence of conjunctivitis (data not shown). However, in one stable a high relative humidity (83%) was noted that might facilitate microbial survival. In the present study, the pigs originated from several piglet producing herds and it should be emphasized that intensive farming systems with the mixing of pigs from several sources also imply increased opportunities for microbial spread among animals of different immunological status. In fact, in one herd a significant relationship was noted between conjunctivitis and the presence of *Chlamydia *spp. when the lower threshold value was applied in the real-time PCR. This might further indicate that the infectious load is important in the development of disease.

Although *Chlamydia *spp. was demonstrated in the ileal sections by immunohistochemistry in the present study, lesions compatible to those described in gnotobiotic pigs [9] were only noted in 50% of the pigs. Several authors speculate that the development of lesions may depend on different factors such as the virulence of the strain, the infectious dose, the route of infection, or the age and the immunological status of the host [1,6,8,10,11,27]. Since young, naive pigs seem to develop lesions in response to an experimental infection [9], host immunity might be induced at an early age in the field [11,28]. It is also possible that co-infections with other presumptive pathogens might act synergistically to exacerbate the lesions or that lesions induced by one microbe might increase the susceptibility to other infections. Several pathogens have been discussed in this respect [3,6,11,13,29,30]. Most of the intestinal lesions described in the present study were shown to be related to infections with *L. intracellularis *or *B. pilosicoli *[17].

Some studies also report the occurrence of mixed infection with several chlamydial species [3,31-33]. In wild boars, *C. psittaci *was the dominating species but *C. suis *and *C. abortus *was also demonstrated by sequencing [31]. In the present study, preliminary data obtained by PCR-RFLP indicated the co-infection by other species [34]. However, sequencing of the amplicons revealed the involvement of *C. suis *only, whereas other bands detected by PCR originated from *E. coli *and un-identified bacterial species. The results underline the difficulties involved in the diagnosis and a large variation in reported prevalences between various detection methods exist [3,15,16,35]. In the present study, the problem was circumvented by the use of an internal probe and a high melting temperature in the real-time PCR, combined with sequencing of the amplicons that assured a high specificity.

## Conclusions

*Chlamydia suis *was found in high prevalences in growing pigs with or without diarrhoea, and in finisher pigs with or without conjunctivitis. Overall, no correlation to clinical signs was detected. However, a correlation was noted between samples with a high degree of infection and the presence of clinical signs. It is possible, that the intensive pig producing systems investigated in the present study might predispose for the transmission and survival of the microbe, thus increasing the infectious load and the risk for disease in the pig. Interestingly, no other species of the *Chlamydiaceae *family were detected, as also supported by the findings in other animal species in Swedish surveys [36].

## Competing interests

The authors declare that they have no competing interests.

## Authors' contributions

SE was responsible for the PCR investigation and sequencing of DNA from the intestinal specimens, analysis and interpretation of these data, and revised the manuscript. CHS made an intellectual contribution by drafting the manuscript and revising it critically. FA was responsible for collecting of the conjunctival swabs, PCR analysis and interpretation of these data, and revised the manuscript. EW was responsible for the immunohistochemical analyses, interpretations of these data, and revised the manuscript. MJ was planning the studies, collected and prepared the intestinal specimens, and was responsible for drafting and writing the manuscript. All authors read and approved the final manuscript.
